# The lipidomes of *C. elegans* with mutations in *asm-3*/acid sphingomyelinase and *hyl-2*/ceramide synthase show distinct lipid profiles during aging

**DOI:** 10.18632/aging.204515

**Published:** 2023-02-13

**Authors:** Trisha A. Staab, Grace McIntyre, Lu Wang, Joycelyn Radeny, Lisa Bettcher, Melissa Guillen, Margaret P. Peck, Azia P. Kalil, Samantha P. Bromley, Daniel Raftery, Jason P. Chan

**Affiliations:** 1 Department of Biology, Marian University, Indianapolis, IN 46222, USA; 2 Department of Environmental and Occupational Health Sciences, University of Washington, Seattle, WA 98195, USA; 3 Northwest Metabolomics Research Center, University of Washington, Seattle, WA 98195, USA; 4 Department of Biology, Juniata College, Huntingdon, PA 16652, USA

**Keywords:** lipidomics, aging, sphingolipid metabolism, *C. elegans*, fatty acid metabolism

## Abstract

Lipid metabolism affects cell and physiological functions that mediate animal healthspan and lifespan. Lipidomics approaches in model organisms have allowed us to better understand changes in lipid composition related to age and lifespan. Here, using the model *C. elegans*, we examine the lipidomes of mutants lacking enzymes critical for sphingolipid metabolism; specifically, we examine acid sphingomyelinase (*asm-3*), which breaks down sphingomyelin to ceramide, and ceramide synthase (*hyl-2*), which synthesizes ceramide from sphingosine. Worm *asm-3* and *hyl-2* mutants have been previously found to be long- and short-lived, respectively. We analyzed longitudinal lipid changes in wild type animals compared to mutants at 1-, 5-, and 10-days of age. We detected over 700 different lipids in several lipid classes. Results indicate that wildtype animals exhibit increased triacylglycerols (TAG) at 10-days compared to 1-day, and decreased lysophoshatidylcholines (LPC). We find that 10-day *hyl-2* mutants have elevated total polyunsaturated fatty acids (PUFA) and increased LPCs compared to 10-day wildtype animals. These changes mirror another short-lived model, the *daf-16*/FOXO transcription factor that is downstream of the insulin-like signaling pathway. In addition, we find that *hyl-2* mutants have poor oxidative stress response, supporting a model where mutants with elevated PUFAs may accumulate more oxidative damage. On the other hand, 10-day *asm-3* mutants have fewer TAGs. Intriguingly, *asm-3* mutants have a similar lipid composition as the long-lived, caloric restriction model *eat-2*/mAChR mutant. Together, these analyses highlight the utility of lipidomic analyses to characterize metabolic changes during aging in *C. elegans*.

## INTRODUCTION

Lipidomic analysis is an emerging field for investigating lifespan regulation and age-related diseases. Lipidomic profiles can be determinants of lifespan and disease conditions [1, 2]. For example, lipid classes including fatty acids (FA), triacylglycerols (TAG), sphingolipids (SL), and phospholipids (PL) have been identified as targets in lipid signatures related to aging [2, 3]. Furthermore, specific signatures are detected in the lipid profiles of those with age-related diseases, such as Alzheimer’s Disease [4–9]. In addition, the abundance of many fatty acid subtypes differs between the youth, elderly, and centenarians [10, 11].

Lipids serve diverse functions and occur in varied locations within cells. The lipid composition of cell membranes can affect aging processes through regulating protein localization, peroxidation, and cell structure. Intracellular lipids, on the other hand, can be involved in signaling, be sources of energy storage, and affect trafficking and organelle functions related to stress responses [12, 13]. For example, the degree of saturation of membrane fatty acids and the levels of polyunsaturated fatty acids (PUFAs) may mediate oxidative damage associated with aging cells [2]. Lipid analyses using model organisms have correlated longevity to elevated PUFAs, as these long carbon chains are more susceptible to oxidative damage [2, 14]. Furthermore, breakdown of lipid droplets that store TAG through lipophagy may release specific fatty acids that mediate the transcriptional regulation of genes involved in stress response and longevity [13, 15, 16]. While TAG levels determine cellular energy storage, total TAGs may have both beneficial and detrimental roles depending on the type of lipid and the organism [17, 18].

Sphingolipid changes are observed in lipidomic profiles associated with age, making it an interesting biomarker for aging [19]; in particular, sphingomyelin (SM) abundance is associated with lifespan in several organisms, including *C. elegans* [20–23]. For example, mutants lacking acid sphingomyelinase (*asm-3*) have longer lifespans [24]. Interestingly, ASM-3 may interact with the DAF-2/insulin-like growth factor receptor, possibly by regulating receptor localization, as loss of *asm-3* increased the nuclear localization of the DAF-16/FOXO transcription factor that is normally inhibited by DAF-2 [24, 25]. Indeed, further evidence supports that sphingolipids make up a major component of microdomains in cell membranes and may mediate protein localization, including receptor localization [26–28]. Interestingly, specific sphingolipid metabolism enzymes, including sphingomyelinase and ceramidase, may have increased activity during aging in rats [29]. However, it is not clear how changes in sphingolipid metabolic enzymes may impact other sphingolipids and larger lipidomic changes.

Many of the metabolic enzymes that mediate lipid synthesis or breakdown have been linked to stress response and aging in organisms [1]. For example, several genes involved with fatty acid elongation (*Elovl5*), desaturation (*Scd5*), and mitochondrial fatty acid synthesis (*Mecr* and *Oxsm*) have been identified as targets of natural selection related to increased lifespan in mammals [30]. In *C. elegans*, fatty acid elongation also seems important for aging [1, 31]. For example, knockdown of *elo-2*, a fatty acid elongase, results in increased lifespan, while knockdown of *elo-5* decreases lifespan. In addition, genetic studies targeting sphingolipid metabolism suggest that specific enzymes may mediate lifespan in worms and flies, and chronological and replicative lifespan in yeast [25, 32–34].

Studies identifying new regulators of aging are critical, given the global rise in an aging population. Lipidomic studies serve as a powerful approach to identify specific metabolic pathways that may contribute to organismal longevity, healthy aging, or pathology. Indeed, lipidomic approaches in long-lived humans and model organisms have provided insight into the lipid changes associated with healthy aging [1, 17, 35]. In particular, work using *C. elegans* have identified age related changes in specific lipids, lipid classes, as well as the ratio of monosaturated to polysaturated fatty acids (MUFA:PUFA ratio) [36, 37]. Here, we examine the lipidomes of animals lacking the sphingolipid metabolism enzymes, *asm-3*/acid sphingomyelinase or *hyl-2*/ceramide synthase, which have previously been shown to have extended and reduced lifespans, respectively, in *C. elegans* [24, 34, 38]. Understanding enzymatic regulation of sphingolipid metabolism is valuable, as changes to sphingomyelins and ceramides impact cell membranes, cell death signaling, and aging [39]. Using *asm-3* and *hyl-2* mutants, we find that each has a unique lipidome compared to wild type, and each shares a similar lipid profile with other short and long-lived *C. elegans* models of aging, respectively. These findings add to our understanding of lipid changes associated with aging.

## RESULTS

### Altering ceramide metabolism affects oxidative stress responses and lipid abundance

**Figure 1:**
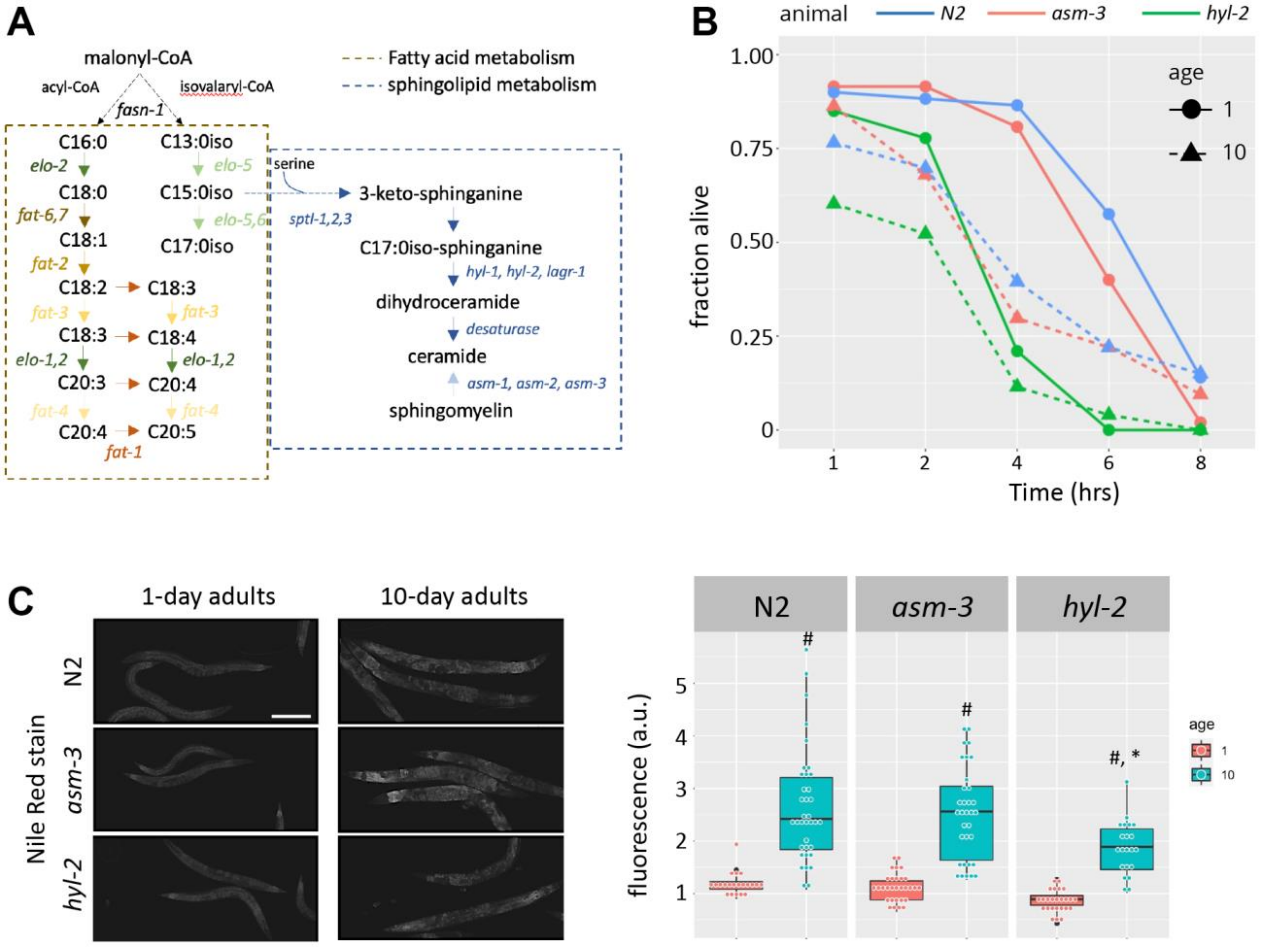
**Loss of *hyl-2*/CER synthase results in poor oxidative stress responses.** (**A**) Diagram showing the lipid pathways and metabolic enzymes producing free fatty acids (FFAs) and sphingolipids (SLs). (**B**) Survival of 1-, 5-, and 10-day old N2 (blue), *asm-3* (red), and *hyl-2* (green) worms treated with 150μM juglone. Worms were treated with juglone in 96 well plates, and survival was determined by a movement response to agitation. Survival curves were analyzed using Kaplan-Meier estimate and pairwise differences were determined using log-rank tests (n=49 for N2 1 day; n=48 for N2 10 day; n=46 for *asm-3* 1 day; n=35 for *asm-3* 10 day; n=28 for *hyl-2* 1 day; n=44 for *hyl-2* 10 day). (**C**) Representative images (left) and fluorescence quantification (right) of Nile Red lipid staining in 1-day and 10-day old N2, *asm-3*, and *hyl-2* animals. ^#^indicates *p*<0.05 compared to 1-day counterpart of the same genotype and *indicates *p*<0.05 compared to N2 counterpart of the same age.

Previous work has shown that *C. elegans* with abnormal ceramide metabolism have altered lifespans and stress response. For example, genetic manipulations leading to the loss of *hyl-2*/CER synthase - which makes ceramide (Figure 1A) - result in shortened lifespans, hypersensitivity to anoxia, and poor survival to heat stress [34, 38, 40]. On the other hand, genetic and pharmacological manipulations that inhibit *asm-3*/acid sphingomyelinase - which converts sphingomyelin to ceramide - increase lifespan [24, 25]. Here, we explored the age-related response of *hyl-2* and *asm-3* mutants to oxidative stress induced by juglone (JG). We found that *hyl-2* mutants at both young and old ages are more sensitive to oxidative stress compared to wildtype (N2) animals. Specifically, survival of *hyl-2* animals exposed to 150μM juglone decreased significantly from wildtype animals at 1 and 10 days of age (Kaplan-Meier estimate and log rank test; *p*=8.5e-11 and *p*=0.01455 respectively), while *asm-3* mutants did not differ from wildtype animals at either age (*p*>0.05 for both; Figure 1B). *hyl-2* mutants showed a similar response to paraquat (PQ) (Supplementary Figure 1).

Given the links between lipid profiles and aging, we next tested whether loss of *hyl-2* or *asm-3* altered lipid abundance using Nile red, a stain primarily for neutral lipids such as triglycerides, and may particularly label lysosomal related organelles [41, 42]. We found that 10-day old animals had greater Nile red staining compared to their 1-day counterparts (*p*<0.001 for all genotypes). Comparing across genotypes showed that 10-day *hyl-2* mutants had significantly less Nile red staining than N2 animals (*p*=9.8e-4) but not at the 1-day timepoint (*p*=0.61); contrary, *asm-3* animals were not statistically different from N2 on either day. These findings suggest that manipulations in ceramide metabolism may produce broad lipid changes in older animals.

### Lipidomes of *hyl-2* and *asm-3* mutants show differential lipid changes

To explore specific lipid changes in *asm-3* and *hyl-2* mutants, we used a lipidomics approach to evaluate changes in specific lipid molecules and classes of lipids. Using shotgun lipidomics by electrospray ionization (ESI) and mass spectrometry, we sampled six independent replicates of 1-, 5-, and 10-day old adult worms of different genotypes. We profiled N2, *asm-3,* and *hyl-2* animals as well as two well-studied mutant models of aging (the short-lived *daf-16*/FOXO mutant and the long-lived, calorically restricted *eat-2*/mAChR mutant). Overall, 701 lipids were identified and analyzed covering phosphatidylcholines (PC), phosphatidylethanolamines (PE), lysophosphatidylcholines (LPC), lysophosphatidylethanolamine (LPE), sphingomyelins (SM), cholesterol esters (CE), ceramides (CER), free fatty acids (FFA), diacylglycerols (DAG), and triacylglycerols (TAG). We detected significant differences in class composition between the different strains and age groups compared to 1-day old N2 adults (Supplementary Figure 2). Interestingly, TAG increased in 10-day old animals compared to 1-day old animals in N2, *daf-16,* and *hyl-2* animals. However, this increase was not observed in long-lived *eat-2* or *asm-3* animals. Overall, we found that our samples had 12.1% FFA, 26.1% TAG, 2.0% DAG, 35.0% PEs, and 16.0% PCs, which is somewhat comparable to that observed in yolk (16.2%, 26.4%, 3.2%, 28.2% and 23%, reference [43]). However, lipid composition is likely to vary by technique and tissue preparation [44].

**Figure 2:**
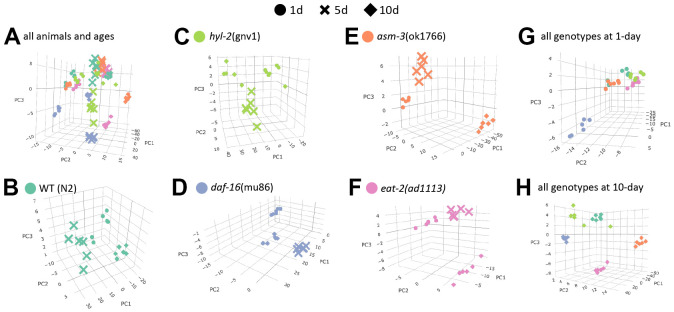
**Age is a major determinant of lipid profiles in *C. elegans*.** Principal component analysis (PCA) graphs show lipid profiles by 1-day (circles), 5-day (cross bars), and 10-day (diamonds). (**A**) PCA graph showing variation all groups, by strain and age. (**B**–**F**) PCA graphs showing variations in age by strain (different colors as indicated). (**G**–**H**) PCA graphs showing variations by 1-day old animals (**G**) and 10-day old animals (**H**).

To visualize how age affects lipidome changes, we first examined how well lipidomes clustered by age or genotype using principal components analysis (PCA). For this analysis, raw concentrations of each lipid were *log_2_* transformed and analyzed. We found that within each genotype, animals from each age group clustered most closely with animals from the same age group (Figure 2A–2F). This suggests that age is a major driver of lipid profiles regardless of genotype. Interestingly, when analyzing the PCA plots by age, genotypes appear to cluster more closely at 1-day, except *daf-16* mutants which appear to cluster away from the rest (Figure 2G, 2H). This visual representation suggests that as animals age, their lipid profile may become more distinct by genotype. Thus, we performed more detailed analysis of specific changes.

**Figure 3:**
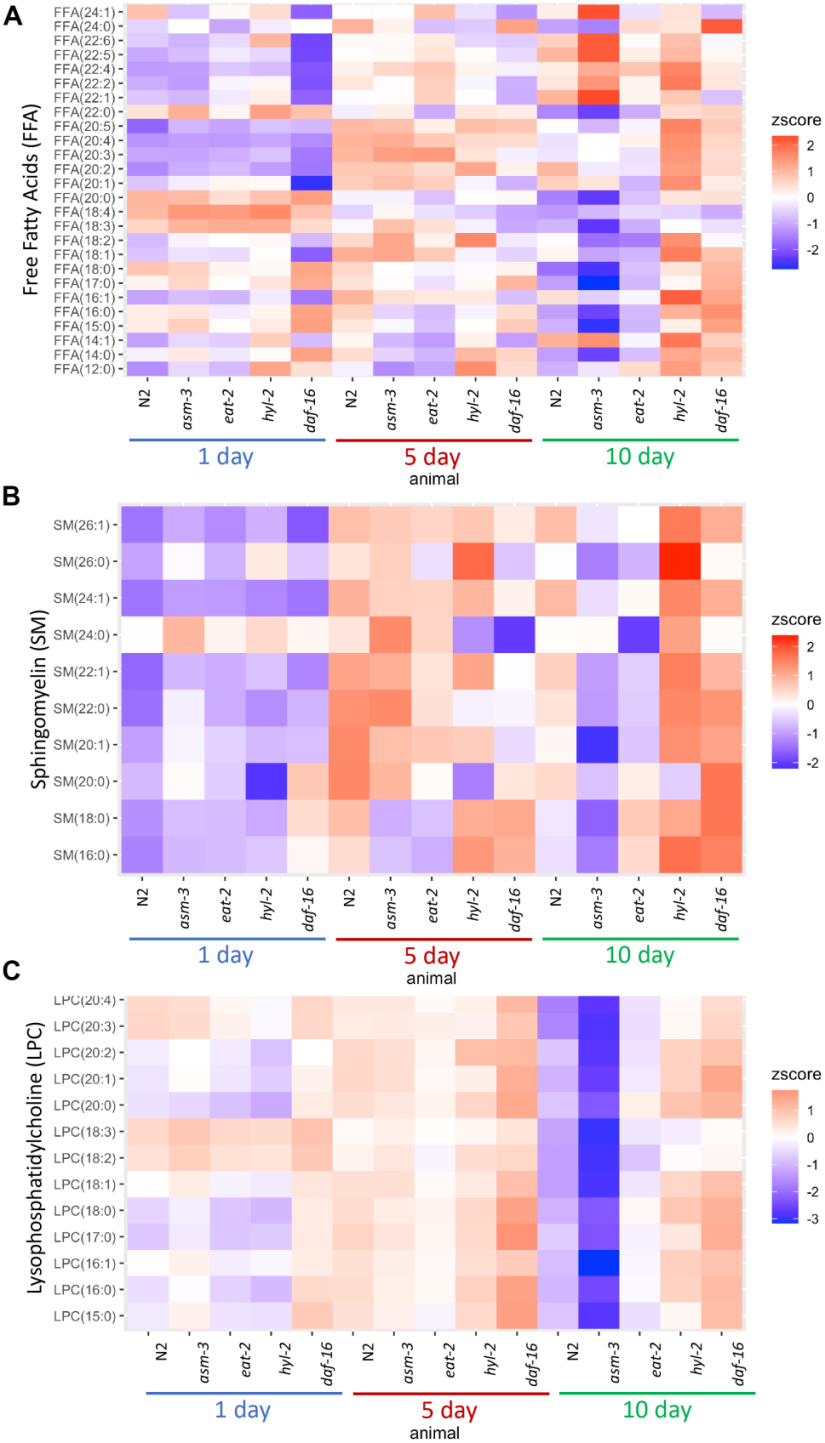
**Lipid profiles vary by age and genotype.** Heatmaps showing average z-score of log2 concentration for (**A**) free fatty acids (FFA), (**B**) sphingomyelins, and (**C**) lysophosphatidylcholines (LPCs). For sphingomyelins, the lipid ID does not include the 18:1 fatty acid chain, and LIPIDMAPS nomenclature is reported in Supplementary Table 11. Wildtype, *asm-3*, *eat-2, hyl-2,* and *daf-16* worms were analyzed at 1-, 5- and 10-days old. For all, the heatmaps show averaged z scores for the six replicates in each group.

Next, we determined whether age affected the lipidome within specific classes of lipids. To do this, we visualized lipid concentrations through heatmaps of free fatty acids (FFA) in N2, *asm-3*, and *hyl-2* mutants (Figure 3A). Wildtype N2 animal show decreases in many shorter chained FFAs, particularly saturated FFA. The largest decreases were in FFA(20:0) (log_2_FC = -2.6, *FDR*=1.60e-15), FFA(18:0) (log_2_FC = -2.5, FDR=2.61e-19) (Supplementary Tables 2, 5). This observation is more pronounced in *asm-3* mutants. However, *hyl-2* mutants show increases in FFA of most types from 1 to 10 days, including many of shorter chained saturated and unsaturated FFAs; this is similar to the short-lived *daf-16* mutant. Interestingly, long-lived *eat-2* and *daf-2* mutants have been shown to have lower amounts of shorter chained fatty acids (C14:0 - C20:0) [6], suggesting that shorter chain FA may contribute to aging. However, very long chain polyunsaturated FFAs increase with age in wildtype animals (Figure 3 and Supplementary Tables 2, 5). The largest changes of FFA from 1 day to 10 day N2 were in polyunsaturated FFA(20:2) (log_2_FC = 2.3, FDR=1.99e-34) and FFA(22:5) (log_2_FC = 1.30, FDR=1.75e-14). Interestingly, *asm-3* mutants exhibit increases in many of the longer chained FFAs at 10 days compared to 1 day (Figure 3A and Supplementary Tables 3, 8).

We then examined sphingomyelin (SM) profiles and found that, in general, SMs increase with age in N2 animals (Figure 3B and Supplementary Figure 3). This was also found by Cutler et al. (2014) that analyzed SM levels from egg to 11-day adults [25]. However, our analysis was different as we did not directly examine the d17:1 iso-sphingoid base that is most prominent in *C. elegans* [45]. Of the SMs we identified, we found that *asm-3* mutants had higher total SMs compared to N2 at 1 day (*p*=0.03) but then was lower at 10 days compared to N2 (*p*<0.00001). In *C. elegans*, there are three acid sphingomyelinases genes (*asm-1, 2, 3*) and an uncharacterized neutral sphingomyelinase (T27F6.6). However, their adult expression patterns are unknown, and therefore, the different enzymes may differentially contribute to sphingolipid metabolism and total sphingomyelin levels throughout the worm’s life. Indeed, *asm-1, asm-2*, and *asm-3* knockdown by RNAi lead to small increases in lifespan independently, and *asm-1* and *asm-2* knockdown by RNAi can further increase lifespan of *asm-3* mutants [24].

Of the SMs we observed, which are more similar to mammalian SMs, the largest increases observed in N2 animals from 1-day to 10-day adults are in several monosaturated sphingomyelins, specifically SM(24:1) (log_2_FC = 2.6, FDR=2.43e-28), SM(22:1) (log_2_FC = 1.9, FDR=4.54e-10) and SM(26:1) (log_2_FC = 1.6, FDR=2.10e-8). Intriguingly, SM(24:1) abundance is low at 1-day in both N2 and *hyl-2* mutants, and increases at both 5 and 10-days. However, in *asm-3* mutants, SM(24:1) abundance is low at 1-day, increases at 5-days, and then decreases again at 10-days. Furthermore, *asm-3* mutants exhibit different 10-to-1 day changes compared to N2; for example, whereas N2 show increases in the saturated sphingomyelins SM(16:0), SM(18:0), SM(22:0), *asm-3* show decreases (Supplementary Tables 3, 8). Interestingly, the saturated sphingomyelins SM(16:0) (log_2_FC=1.9, FDR=2.38e-8), SM(18:0) (log_2_FC=1.3, FDR=0.026), and SM(22:0) (log_2_FC=0.47, FDR=0.0293) show even greater increases from 1 to 10 days in *hyl-2* mutants than N2. Given the role of SMs on membrane fluidity, these age dependent changes may affect membrane properties in *asm-3* and *hyl-2* mutants that affect cell physiology.

**Figure 4:**
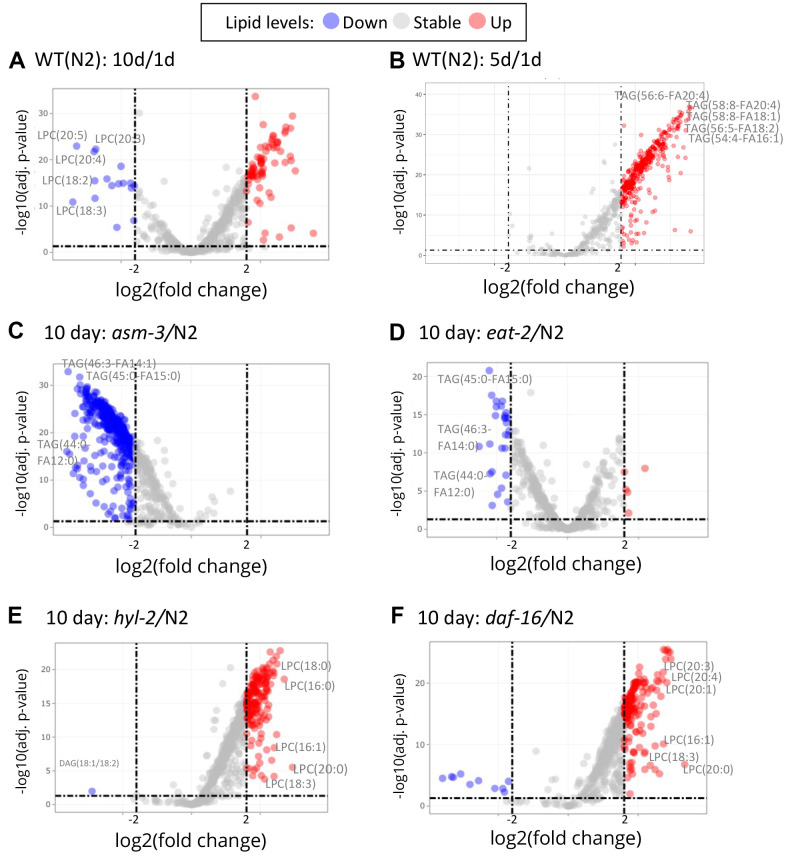
**Volcano plots show that specific lipids change by age and genotype.** Log_2_ fold changes between groups are graphed by adjusted P values. Red indicates a greater than 2 log_2_ fold increase of specific lipid, and blue indicates greater than -2 log_2_ fold decrease (dashed lines). (**A**) Comparison of 10-day old N2 animals versus 1-day old N2 animals (10d/1d). 10-day old N2 animals have many decreased LPCs compared to 1-day. (**B**) Comparison of 5-day old N2 animals versus 1-day old N2 animals. (**C**) Comparison of 10-day old *asm-3* animals versus 10-day old N2 animals. (**D**) Comparison of 10-day old *eat-2* animals versus 10-day old N2 animals. (**E**) Comparison of 10-day old *hyl-2* animals versus 10-day old N2 animals. (**F**) Comparison of 10-day old *daf-16* animals versus 10-day old N2 animals.

To explore specific lipid changes associated with age, we next examined fold changes of each lipid molecule using volcano plots, plotting their concentration to their false discovery rate (FDR, or adjusted *p* value). Those lipids with greater than a 4-fold change (log_2_ FC > 2) are highlighted in Figure 4 (and Supplementary Tables 2–9). First, we examined changes in 10-day old N2 animals compared to 1-day old animals (10d/1d; Figure 4A); many of the lipids that are lower at 10 days compared to 1 day were LPCs, whereas many of the lipids that were higher at 10 days were triacylglycerols (Figure 4A). When examining changes in 5-day old N2 animals compared to 1-day animals, we observed similar changes in triacylglycerols, but not LPCs (Figure 4B). Next, we compared lipidomes of 10-day old animals of *hyl-2, asm-3, daf-16,* and *eat-2* backgrounds to that of wildtype 10-day animals (Figure 4C–4F). Strikingly, the lipid changes in *asm-3* to N2 were similar to that of the long-lived *eat-2* to N2. Most of the highly significant changes were found to be lower levels of TAGs. Indeed, *eat-2* animals exhibit reduced TAG levels by Nile Red, Oil red O, and Sudan black staining, as well as gas chromatography [46, 47]. On the other hand, the profile of lipid changes in *hyl-2* to N2 was similar to that of the short-lived *daf-16* to N2. Both 10-day old *hyl-2* and *daf-16* showed increases in LPCs compared to 10-day wildtype, sharing many common LPCs including LPC(16:1), LPC(18:3) and LPC(20:0).

Given the changes in LPCs, we visualized differences in LPC levels across all animals in a heatmap (Figure 3C). We find that *asm-3* and *eat-2* show normal levels of LPCs at younger ages, but have much lower levels of LPCs at 10-days old. On the other hand, *hyl-2* and *daf-16* show increased LPC levels at younger and older ages. In humans, elevated levels of LPCs in circulating low density lipoprotiens (LDLs) are associated with disease [48], and elevated LPC levels are observed in stress conditions, particularly inflammatory disease [49].

### Saturated and unsaturated fatty acid changes in *hyl-2*/CER synthase and *daf-16*/FOXO mutants

**Figure 5:**
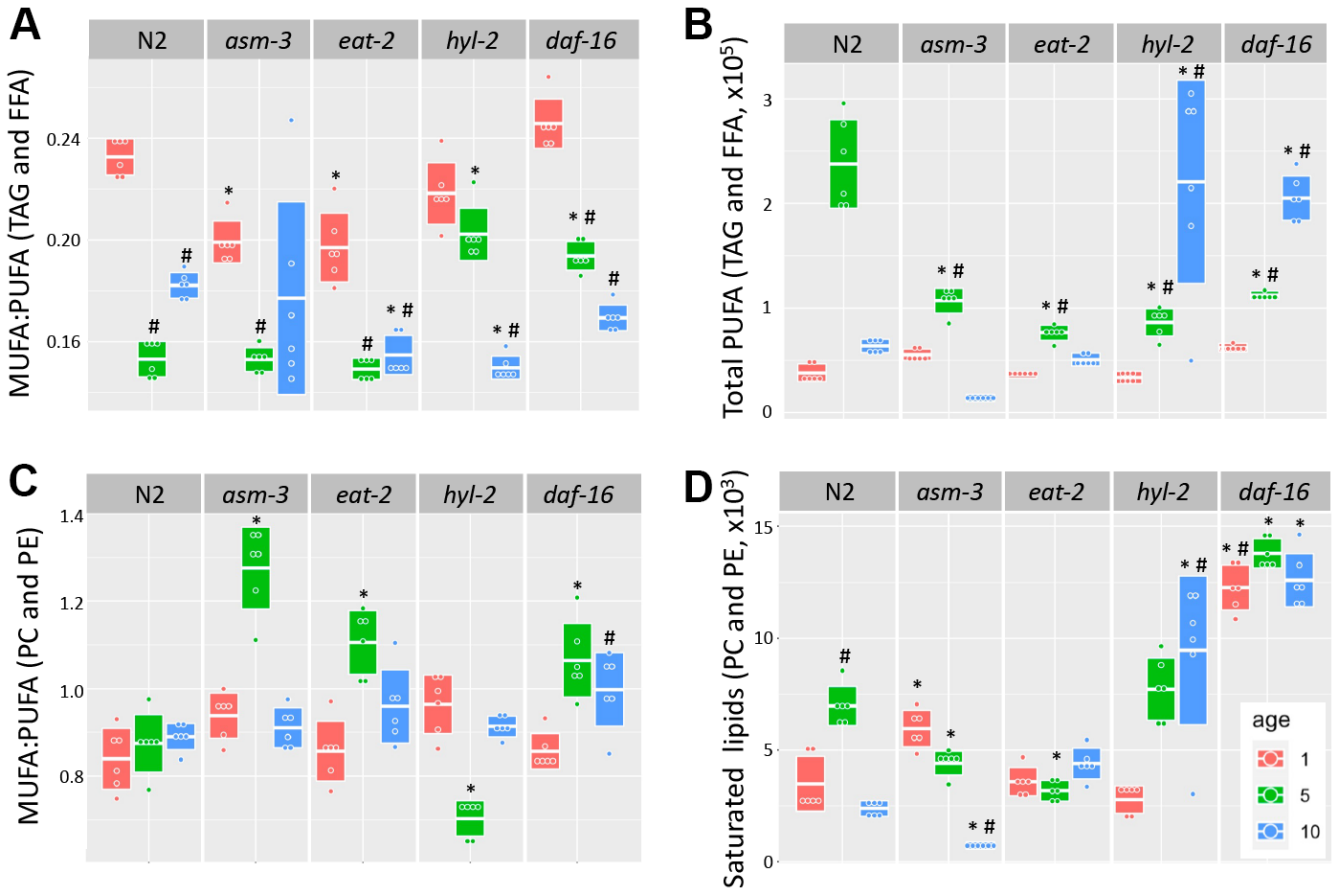
**Analysis of acyl chain saturation during young and old *asm-3* and *hyl-2* mutants.** Chain saturation was analyzed and compared between 1-day (red), 5-day (green), and 10-day (blue) old N2, *asm-3*, *eat-2*, *hyl-2*, and *daf-16* animals. Total (**A**) MUFA:PUFA ratios in TAG and FFA, (**B**) total polyunsaturated chains in in TAG and FFA, (**C**) MUFA:PUFA ratios in PC and PE, and (**D**) saturated PC and PE were summed for each group (n=6). Boxes in graphs represent the middle quartile for the data points in each group. For all, ^#^ indicates *p*<0.05 compared to 1-day counterpart and * indicates *p*<0.05 compared to N2 counterpart.

Fatty acid chain desaturation has been of interest in aging because polyunsaturated fatty acids (PUFAs) may be more susceptible to oxidation and cellular damage, suggesting that high levels of PUFAs may be detrimental to health. Indeed, older animals have a decreased monosaturated fatty acid to polyunsaturated fatty acid (MUFA:PUFA) ratio, suggesting that there are more PUFAs, or less MUFAs, with age; in addition, long-lived animals generally have a higher MUFA:PUFA ratio compared to short-lived animals [35, 36, 50]. We analyzed TAG and FFA and found that the MUFA:PUFA ratio declined in older ages for all genotypes (Figure 5A). However, when we analyzed total PUFAs, we found that both *hyl-2* and *daf-16* mutants had higher amounts of PUFAs than 10-day N2 animals (Figure 5B, 3.46 fold and 3.22 fold, p<1.0e-7 respectively). Both *hyl-2* and *daf-16* had PUFA levels at 1 day comparable to N2, but PUFA levels continued to increase significantly at each timepoint. Both *asm-3* and *eat-2* had non-significant changes at 10 days compared to N2. The changes in MUFA:PUFA ratio in older animals was not observed when we analyzed saturation levels in PC and PE (Figure 5B). However, we did find that *hyl-2* and *daf-16* mutants also had increased amounts of saturated lipids found in PC and PE (Figure 5D, 3.96 fold, and 5.28 fold increase respectively; *p*<1.0e-7 for both).

**Figure 6:**
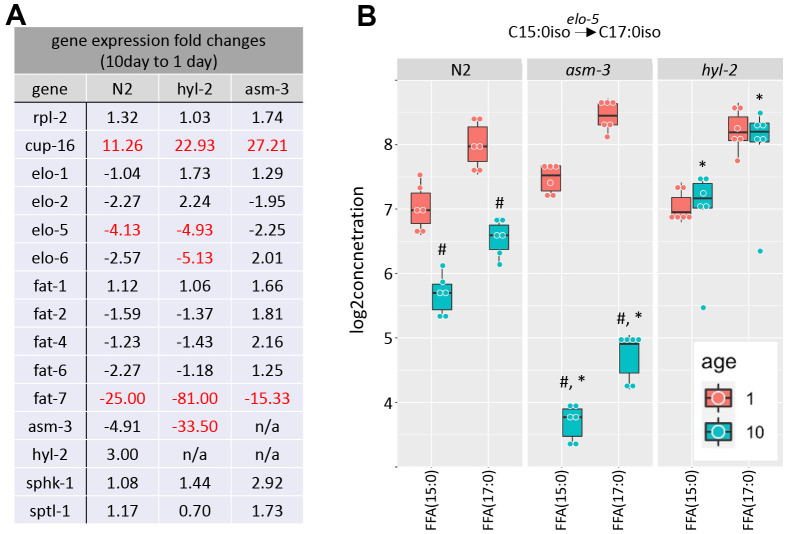
**Saturated and polyunsaturated fatty acids metabolism.** (**A**) Table of gene expression for fatty acid for elongases and desaturates examined in 1-day and 10-day old N2, *asm-3,* and *hyl-2* animals by quantitative PCR. Data are represented by 2^-ddCT^ fold change of 10-day N2 compared to 1-day values (n=3 for each group, red indicates fold changes that are *p*<0.01). The reference gene was *rps-2*, and *rpl-2* and *cup-16* are control genes known to remain unchanged and increase, respectively, in older animals. (**B**) Log_2_ concentrations of FFA(15:0) and FFA(17:0) are shown at 1- and 10-day old N2, *asm-3*, and *hyl-2* animals (n=6 for each group). For all, ^#^indicates *p*<0.05 compared to 1-day counterpart of the same genotype. *indicates *p*<0.05 compared to N2 counterpart of the same age.

Metabolic enzymes that produce elongated and desaturated fatty acids (Figure 1A) have been implicated in lifespan regulation [1, 13, 17, 31, 50]. Thus, we next explored whether aging affects gene expression of key enzymes in fatty acid lipid metabolism. Fatty acid metabolism starts with the fatty acid synthase FASN-1, which modifies malonyl-CoA to generate either C16 straight chain fatty acids by using Acetyl-CoA, or C13iso-branched fatty acid by using isovalaryl-CoA. From there, C16:0 can be elongated by fatty acid elongases or desaturated by fatty acid desaturases. We examined by qPCR whether elongases or desaturates are regulated by age or by mutations in *hyl-2* and *asm-3*. We found that two fatty acid elongases (*elo-1* and *elo-2*) did not change when comparing 1-day and 10-day old animals of any genotype (Figure 6A and Supplementary Table 10). When then analyzed fatty acid desaturases in the pathway (*fat-1*, *fat-2, fat-4, fat-6, and fat-7*). We found that 10-day old animals of all genotypes have very low expression of *fat-7*, which is a FA desaturase that converts FFA(18:0) to FFA(18:1). We did not observe changes in *fat-6* expression or other fatty acid desaturases examined.

C13:0iso-branched fatty acid leads to the synthesis of monomethyl branched chain fatty acids (mmBCFA), which are involved in growth and survival [51, 52]. ELO-5 and ELO-6 specifically transfer acyl groups to C13iso and C15iso fatty acids [51, 53]. We examined whether there were gene expression changes in *elo-5* and *elo-6* in 1- and 10-day old N2, *hyl-2*, and *asm-3* animals. Interestingly, *elo-5* showed significant decreases in wildtype and *hyl-2* animals, and non-significant changes in *asm-3* animals (Figure 6A). *hyl-2* further shows significant downregulation of *elo-6*. The downregulation of *elo-5* in N2 and *asm-3* animals may contribute to the lower levels of FFA(17:0) (Figure 6B). However, it is not clear why *hyl-2* mutants exhibit low expression of *elo-5* and *elo-6* despite sustained levels of FFA(17:0). It is possible some feedback mechanisms exist to decrease their expression, or that early steps in mmBCA synthesis are increased to affect sphingolipid metabolism in *hyl-2*. Indeed, many sphingomyelin species are higher in 10-day old *hyl-2* mutants (Figure 3B) compared to both 1-day *hyl-2* or N2 animals.

We found that the lipid levels of the FFA(15:0) and FFA(17:0) is increased in 10-day *hyl-2* (log_2_FC=1.28, p=1.66e-10; Figure 6B) and *daf-16* (log_2_FC=2.55, p=2.50e-23) mutants compared to N2. On the other hand, *asm-3* animals showed decreases in FFA(15:0) and FFA(17:0) (log_2_FC=-1.98, p=1.10e-18) (Figure 6B). We were interested in whether the differences in these chains are also observed in triacylglycerols (TAGs). When analyzing FA15:0 and FA17:0 chains that are part of TAGs in *asm-3* animals, we found that they also exhibited decreased TAGs containing FA15:0 and FA17:0 at 10-day compared to 1-day; *hyl-2* mutants showed a general increase in in these TAGs compared to N2, but not in either specific day (Supplementary Figure 4). Although our lipidomics detection could not identify the iso-branched species specifically, this suggests that *hyl-2* might have higher mmBCFAs at older ages, whereas *asm-3* mutants have lower.

To determine whether sphingolipid metabolism itself is altered by age, we examined key enzymes in the production of sphingolipids from mmBCFAs. The mmBCFA C15iso and C17iso fatty acids can be made into C17iso-sphinganine or its precursors through serine palmitoyl transferase (*sptl-1*) in *C. elegans* (Figure 1A) [54, 55]. We found that *sptl-1*/serine palmitoyltransferase does not change with age or strain (Figure 6A). The addition of fatty acid to C17iso-sphinganine to make dihydroceramide is mediated by *hyl-2*, as well as other ceramide synthases (Figure 1A). *hyl-2* specifically transfers fatty acids of shorter chains (C20-22) [40]. We further explored gene expression changes of *hyl-2* and *asm-3* itself, and found that *asm-3* showed a trend to decrease in N2 animals at 10-days compared to 1-day (Figure 6A). However, we observed that, in general, *asm-3* had low expression in all genotypes at 10-days. Interestingly, there was a large 10-day to 1-day decrease in *asm-3* expression observed in *hyl-2* animals. Thus, it is possible that *hyl-2* animals, and old animals in general, have reduced breakdown of sphingomyelin at older ages that contribute to their poor stress response and reduced lifespan. However, given that *asm-3* mutants have increased lifespan, it is not clear how reduced *asm-3* expression at later ages may specifically modify aging processes differently than complete knockouts. Together, these data support a model where increased abundance of sphingolipid precursors and sphingomyelin may contribute to poor aging phenotypes. Indeed, our findings support lipidomic analyses of human longevity suggesting that centenarians upregulate mechanisms to upregulate sphingomyelins to ceramide-containing glycosphingolipids [56, 57].

## DISCUSSION

Ceramide synthase and sphingomyelinase are enzymes in sphingolipid metabolism that impact sphingomyelin and ceramide levels and aging. We examined lipidomes of mutants for these sphingolipid metabolism enzymes in *C. elegans* and observed lipid changes that may begin to elucidate how sphingolipid alterations might alter broad lipid composition. We found that *hyl-2*/CER synthase mutants have poor stress response and have many similar lipidomic changes as the short-lived *daf-16*/FOXO mutant. These include increased total PUFAs and increases in specific LPCs. Conversely, *asm-3*/acid sphingomyelinase mutants have slight resistance to oxidative stress and have lipidomic changes more similar to the long-lived *eat-2*/mAChR model. Further analyses of specific lipid changes may provide insight into how these sphingolipid mutants may mediate stress response and aging. As lipid composition changes with age, better understanding of lipid signatures in mutant models may become useful for aging studies.

### Sphingolipid metabolism changes associated with aging

Sphingolipids are gaining attention in their roles in aging but have diverse and complex functions in cells. Sphingomyelins (SM) are among the most prevalent lipids found on cell membranes, and they influence membrane fluidity and cell signaling. We found that most SMs increase with age in general, and *asm-3*/acid SMase mutants had differing SM profiles than N2. Specifically, 10-day old wild type animals have increased longer chained SMs. While our study did not examine the more abundant d17:1 sphingoid species of SM, our finding supports previous studies of SM changes with age, which show that wildtype animals generally have greater SM elongation (increase C22:0 and C24:0 in 11-day adults compared to 3-day adults) and desaturation (less C18:0) [25]. Longer acyl chained SM create interdigitating acyl chains, which can reduce membrane mobility, and may contribute to the decrease in membrane fluidity observed in aging animals and senescent cells [58–60]. Interestingly, blood serum sphingomyelin is elevated with age and is associated with Alzheimer’s disease [61]. In addition, in the Baltimore longitudinal study, Mielke et al. (2014) found that most SMs increase with age, particularly in female subjects; however, some longer chained saturated SMs (C20:0, C22:0 and C24:0) increase in early aging but may decrease with late age (>70years old) [22]. Saturated sphingomyelins also appear correlated with obesity and insulin resistance [62].

Why might changes in sphingolipid metabolism cause alterations in adult stress response and lifespan? Ceramides, which can be produced by synthesis (ceramide synthases) or recycling through sphingomyelin (sphingomyelinases), have been found to be important for anoxia, autophagy, mitochondrial stress response, locomotion and others in *C. elegans* [38, 40, 63, 64]. Thus, it is likely that altered activity of enzymes such as ceramide synthases and acid sphingomyelinases serve broad roles in aging physiology. More studies showing direct impact of lipid changes will help elucidate how sphingolipids may mediate cellular processes associated with aging. For example, Wang et al. (2021) show that glucosylceramides mediate clathrin binding to autophagolysosome for lysosome recycling. Furthermore, SM levels and ASM-3 function have been associated with insulin receptors signaling, suggesting that loss of specific SM may reduce or alter receptor abundance or localization [24, 61, 65]. Indeed, Kim and Sun (2012) show that mutations or pharmacological inhibition decreasing *asm-3* function increases lifespan and possibly through altered localization of the DAF-2/insulin-like receptors, or downstream targets, that are associated with stress response and aging in worms. However, the specific sphingomyelins species were not examined in these studies, and further characterization of specific carbon length and saturation may provide greater insight into mechanisms of sphingomyelin regulation of aging. On the other hand, studies on Drosophila *cpes* mutants, which have reduced ceramide phosphoethanolamine (CPE) – a structural analog of mammalian sphingomyelin – show that CPE has a positive correlation to lifespan increase; specifically, the presence of CPE on glial membranes alters circadian rhythms, glutamate homeostasis and shortens lifespan [66]. Furthermore, sphingolipids on synaptic vesicles have been shown to mediate SNARE proteins and exocytosis [67]. Thus, changes in SMs may alter specific organelle and cell membrane composition that impacts local protein-lipid interactions.

Interestingly, we found that *asm-3* gene expression decreases with age, suggesting that sphingomyelin breakdown and broad lipid profiles may change during aging. Indeed, examining SM show that SMs increase with age, and are higher in short-lived animals. However, lipid profiles may be highly specific to the organism, as higher sphingolipid saturation overall was also observed in other long-living species [3]. It is worth noting that there are also race specific effects, as higher levels of sphingomyelins are observed in African Americans and Hispanics when compared to Caucasians [22, 68]. Thus, the changes in SMs are complex, and may involve specific lipids, interactions with other factors and protein functions. Though not definitive, studies suggest that specific sphingomyelins, and enzymes that alter carbon length or saturation, may play important roles in maintaining membrane sphingolipid composition important for many receptors or proteins associated with aging.

### Fatty acids associated with fat storage and lipid oxidation

Free fatty acids (FFA) serve roles in both fat storage and membrane lipid composition. Fat storage is important for cellular energy and can change with age, but there is not a direct correlation between lipid levels and lifespan [69]. The dietary restriction mutant model *eat-2* has lower fats in storage whereas the insulin signaling model *daf-2*(e1370) has high levels of TAG; yet both are long-lived [37, 41, 69–72]. Furthermore, effects may even be genotype dependent, as not all *daf-2* mutant alleles share the higher TAG phenotype (Perez and Van Gilst 2008). However, higher TAG have been proposed to contribute to the longer lifespans of animals due to the greater reserve of stored fats for energy utilization in late life [13]. In other model organisms such as yeast, deletion of triglyceride lipases results in increased lipid accumulation as well as increased lifespan [18, 73].

Lipid droplets (LD) contain stored triacylglycerols, and these TAGs are broken down by enzymes that mediate lipophagy to make free fatty acids [16]. This process may involve autophagy genes as well as the lipase *lipl-4* in *C. elegans* [74, 75]. Indeed, *lipl-4* is required for the long lifespan phenotype of *daf-2* mutants [76]. Interestingly, glycosphingolipids have recently been shown to mediate clathrin-dependent lysosome formation during autophagy, and increased autophagy in the long-lived *hyl-1;lagr-1* ceramide synthase mutants (which produce longer chained ceramides) is abolished by loss of *atg-12* [34, 63]. Thus, it is intriguing to speculate that changes in sphingolipid metabolism may mediate membrane dynamics that facilitate lipophagy. Indeed, acid sphingomyelinase (aSMase) is found in lysosomes, and *asm-3*/acid SMase is involved in lipid storage in embryos and may have roles in general lipid metabolism [77, 78]. However, Nile Red staining, which might more specifically label lysosomal lipids, did not show broad changes in lipids levels.

Fatty acids also make up more complex lipids that are part of cell and organelle membranes. The oxidative theory of aging suggests that increased lipids, such as polyunsaturated fatty acids (PUFAs), are more susceptible to oxidation and can lead to cell damage during aging [79, 80]. Indeed, elevated levels of PUFAs appear to increase oxidation and cell damage, and low PUFA levels correlate to longevity in mammals and worms [10, 50]. In *C. elegans*, PUFAs comprise 46% of total fatty acids of phospholipids but only 12% of triacylglycerols, suggesting that a greater percent of fatty acids is found in phospholipids than triacylglycerols [31]. We found that *hyl-2/*CER synthase and shorter lived *daf-16* have higher amounts of many PUFAs, and this may contribute to their poor lifespan and healthspan in these animals. Many long-lived worms also have increases in MUFA:PUFA ratios, suggesting that there are less polyunsaturated fatty acids available for oxidative damage. Indeed, mutations in Δ9 desaturases (*fat-5*, *fat-6*, and *fat-7*), which produces PUFAs in *C. elegans*, or supplementation of the PUFA eicosapentaenoic acid (EPA) shortens lifespan [50]. However, ω-6 PUFAs has also been shown increase autophagy and extend lifespan [81]. This discrepancy is observed elsewhere as a study showed that centenarians increase ω-3 to ω-6 PUFAs [82]. Furthermore, long-lived *daf-2* mutants have lower expression of *fasn-1*, a fatty acid synthase important for the initial steps of fatty acid production [83]. This may suggest a downregulation of the metabolic processes associated with fatty acid production, elongation, or desaturation mediates the effects of lipid metabolism on aging.

Phylogenomic studies have also identified evolutionary targets that link metabolic enzymes to longevity and include genes with functions in fatty acid elongation and desaturation [30]. We found that age decreases the expression of *elo-5*, which produces C15iso lipids and is important for development [51]. Interestingly, the long-lived phenotype of *daf-2* is somewhat dependent on *elo*-*5* [84]. We found that *hyl-2* and *daf-16* have increased levels of longer chained PUFAs at 10-days, compared to wildtype animals, suggesting that fatty acid metabolism in these animals may be increased. Shmookler-Reis (2011) also suggests that elongase activity negatively correlates with aging; specifically, the combined regulation for increased Δ9-desaturase activity to generate MUFAs, and decreased activity of downstream Δ5-desaturases that generate PUFAs, may correlate with longevity.

Different methodologies to lipid extractions can yield changes in lipid composition. We note that our protocol called for thawing frozen samples prior to lipidomics sample preparation. This freeze and thaw have been previously done on *C. elegans* for lipidomics [36, 85], and our quality control does not suggest degradation of any particular class of lipids. However, others have prepped from frozen C. elegans extracts directly [31]. Different tissue treatment conditions can damage tissues and result in elevations in FFA within samples [44]. Thus, total FFA and LPC levels may be impacted by sample preparation methods, although we do not suspect changes in relative values between genotypes emphasized in this analysis.

### Lysophosphatidylcholines are increased in short-lived animals

High levels of LPCs may mediate cell stress. We found that LPCs were more abundant in short-lived mutants, *hyl-2*/CER synthase and *daf-16*/FOXO. LPCs can increase reactive oxygen species in aortic endothelial cells [86]. In addition, osmotic stress was shown to increase the production of LPC through *daf-16*, and that LPC(18:0) specifically caused osmotic stress mediated embryonic lethality [87]. Furthermore, *daf-2*/insulin-like receptor mutants exhibit 50% lower levels of LPCs compared to the wild type, including LPC(18:0) and PUFA containing LPCs (20:5, 20:4, and 20:3) [37]. In addition, Rebaudioside A, a sweetener that decreased ROS accumulation and improved oxidative stress response, caused decreased levels of LPC(20:4) and LPC(20:5) [88]. Intriguingly, human studies also show that LPC(20:3) and LPC(20:4) are lower in elderly [19]. On the other hand, others have shown that low levels of LPCs have also been linked to poor mitochondrial function [1]. Together, these data may suggest that regulation of LPC content may mediate mitochondrial function and oxidative stress in animals, and that low or high LPCs may affect healthspan or lifespan.

In summary, analyzing lipidomes of models of aging has the potential to identify cellular processes and signaling pathways important for animal stress response and aging. We found here that *C. elegans hyl-2*/CER synthase mutants had a 10-day lipid profile that mirrored shorter lived *daf-16*/FOXO mutants, which included elevated PUFAs and LPCs. Age caused increased sphingomyelin levels, particularly in short-lived animals. This may suggest that the regulation of sphingolipid metabolism may mediate changes in cell structure and function important for healthy aging. Future studies connecting lipidomic changes in sphingolipid metabolism mutants to mechanistic changes in cells of mutant models will be important next steps to better understanding the roles of sphingolipids in aging.

## MATERIALS AND METHODS

### *C. elegans* strains

All *C. elegans* were grown on nematode growth media (NGM) at 20° C methods (Stiernagle 2006). Plates were seeded with OP50 *E. coli*. For lifespan and aging studies, NGM plates were supplemented with 50μM 5-Fluoro-2′-deoxyuridine (FUdR, Alfa Aesar). The following strains were provided by the CGC, which is funded by NIH Office of Research Infrastructure Programs (P40 OD010440): *hyl-2(gnv1), asm-3(ok1744)*, *eat-2(ad1113),* and *daf-16(mu86)*. The wildtype strain used was N2 Bristol. All strains were outcrossed at least 4x.

### Oxidative stress assays

For stress assays, *N2*, *hyl-2(gnv-1),* and *asm-3(ok1744)* animals were synchronized via egg lay on NGM plates seeded with OP50 *E. coli*. When progeny reached the L4 stage, animals were washed with M9 and transferred to NGM/OP50 plates supplemented with 50μM FUdR. For acute juglone assays, animals were grown to 1-, 5-, and 10-day adults. At these ages, approximately 50 adult worms per genotype were transferred to 150μM juglone (Sigma Aldrich) in M9 by placing 10-15 animals per well for 4 wells, in a 96 well plate [89]. Worms were scored for survival every two hours for 8 hours. Animals were considered dead if they did not respond to agitation. Data was analyzed using Kaplan-Meier estimates and log-rank tests, with Bonferroni correction, in the R statistical package *survival* and *survminer*.

### Nile red staining and imaging

To generate a synchronous population of worms, adult N2, *hyl-2(gnv1),* and *asm-3(ok1744)* animals were allowed to lay eggs for 4-6 hours on NGM plates seeded with OP50 *E. coli*. When progeny reached the L4 stage, they were moved onto OP50 *E. coli* seeded NGM plates supplemented with 50μM FUdR. At 1-day or 10-days after the L4 stage, animals stained with Nile Red as previously described previously [42]*.* Briefly, animals were washed with PBST, incubated in 40% isopropanol of 3 minutes, stained with Nile Red working solution (30μg/mL 40% isopropanol) for 2 hours, in the dark, shaking at room temperature. After incubation, worms were washed again with PBST in the dark for 30 minutes and worms were moved to a slide for imaging. Worms were imaged using an ECHO Revolve R4 microscope equipped with Olympus UPlan Fluorite objectives in the FITC/GFP channel. Images were then analyzed using ImageJ version 1.53e [90]. Whole worm fluorescence were used for statistical analysis, accounting for background fluorescence, and values were analyzed by two-way ANOVA and Tukey post-hoc tests in the R Statistical Package.

### RNA isolation and real time quantitative polymerase chain reaction

N2, *hyl-2(gnv1),* and *asm-3(ok1744)* animals were synchronized using a standard egg prep protocol and distributed onto NGM plates supplemented with OP50. Once animals reached the L4 stage, they were transferred to NGM plates supplemented with 50μM FUdR and seeded with OP50*.* Approximately 500 worms per biological replicate were collected at 1 day and 10 days past the L4 stage and flash frozen using liquid nitrogen. RNA was isolated using TRIZOL (Sigma Aldrich) and chloroform extraction and then stored at -80° C. The absorbance at 260/280nm was measured using a NanoDrop Spectrophotometer (Thermo Fisher Scientific) to determine the purity of the RNA. cDNA was generated from ~500ng/μL of total RNA using the QuantiNova Reverse Transcription Kit (Qiagen). In the first step of the cDNA synthesis, all RNA was treated with a gDNA removal step to remove gDNA contamination. Quantitative, real-time polymerase chain reaction was performed in triplicate using the QuantiNova SYBR Green PCR Kit and Rotor Gene Q (Qiagen). mRNA fold changes were calculated using ddCT method (Wu, Tian et al. 2020). mRNA levels of the genes of interest were compared with the mRNA levels of the reference gene *rps-2.* Gene expression by qPCR was repeated with biological replicates and three technical replicates. All qPCR primers (Supplementary Table 1) were designed to be intron spanning, and one primer from each set was intron bridging to reduce the likelihood of gDNA amplification. Data were analyzed by two-way ANOVA and Tukey post-hoc tests in the R Statistical Package.

### Sample preparation for lipidomics

Approximately 1000 *C. elegans* worms were synchronized by treating gravid adults with an alkaline hypochlorite solution (bleach/NaOH). Worms were then grown to L4 stage and transferred to NGM agar plates containing 50μM FUdR. Worms were then collected after 1, 5, and 10 days. Six replicate samples of each group were then sent for lipidomics analysis at the Northwest Metabolomics Research Center (University of Washington).

*C. elegans* samples were prepared as described (Hanson et al., 2020). Briefly, frozen worm samples were thawed and vortexed; 250μL of worm slurry was transferred to a borosilicate glass culture tube (16 × 100 mm). Next, 250μL of water, 1 mL of methanol, and 450μL of dichloromethane were added to all samples. Then, 25μL of the isotope labeled internal standards mixture were added and incubated at room temperature for 30 min. Next, another 500μL of water and 450μL of dichloromethane were added to the tube and centrifuged at 2500×g at 15° C for 10 min. The bottom organic layer was transferred to a new tube and 900μL of dichloromethane was added to the original tube for a second extraction. The combined extracts were concentrated under nitrogen and reconstituted in 250μL of the running solution (10mM ammonium acetate in 50:50 methanol:dichloromethane).

### Mass spectrometry

*C. elegans* samples were analyzed as described by [91] and completed at the Northwest Metabolomics Research Center. Briefly, Quantitative lipidomics was performed with the Sciex Lipidyzer platform consisting of a Sciex QTRAP® 5500 mass spectrometer equipped with SelexION® for differential mobility spectrometry (DMS). 1-propanol was used as the chemical modifier for the DMS. Samples were introduced to the mass spectrometer by flow injection analysis at 8 μL/min. Each sample was injected twice, once with the DMS on (PC/PE/LPC/LPE/SM), and once with the DMS off (CE/ CER/DAG/DCER/FFA/HCER/ LCER/TAG). The lipid molecular species were measured using multiple reaction monitoring (MRM) and positive/negative polarity switching. A total of 1070 lipids and fatty acids were targeted in the analysis. Using 54 internal standards previously used and described in [92], quantities (in μmol/g) of each lipid species could be calculated. Data were acquired and processed using Analyst 1.6.3 and Lipidomics Workflow Manager 1.0.5.0.

### Data processing and statistical analyses

Statistical analysis was carried out using R (version 3.6.0). The targeted lipidomic assay was designed to detect 1070 lipid species. Data were in concentration per worm weight (nmol/g of worm tissue (μM)), and therefore no further normalization was performed prior to statistical analysis. Only lipids in which missing data across all groups were less than 20% were included in further analysis. Out of the possible 1071 lipid species that the assay could detect, 701 lipids passed these filtering criteria and were included in further analysis. We used a quantile regression approach for the imputation of left-censored missing data (QRILC), which has been suggested as the favored imputation method for left-censored MNAR data [93]. This was implemented in the R imputeLCMD package.

We fit linear models to the lipidomic data using the Bioconductor limma package [94] to assess the difference in abundance between experimental groups. The limma package uses empirical Bayes moderated statistics, which improves power by ‘borrowing strength’ between features in order to moderate the residual variance [95]). We selected lipids with a false discovery rate (FDR) of 5%.

In the heatmaps, z-scores are calculated by adjusting the data, by feature, to have a mean of zero and a standard deviation of 1. The heatmaps were generated using the R statistical package. For direct comparisons of specific lipids between strains, log_2_ concentrations were compared between samples using a two-way ANOVA and Tukey post hoc analyses in the R statistical package.

## Supplementary Material

Supplementary Figures

Supplementary Table 1

Supplementary Table 2

Supplementary Table 3

Supplementary Table 4

Supplementary Table 5

Supplementary Table 6

Supplementary Table 7

Supplementary Table 8

Supplementary Table 9

Supplementary Tables 10 and 11
