# Supplementary material for: The lipidomes of C. elegans with mutations in asm-3/acid sphingomyelinase and hyl-2/ceramide synthase show distinct lipid profiles during aging
Source: Aging (Albany NY). 2023 Feb 13;15(3):650–74. doi: 10.18632/aging.204515 (PMC9970312; doi:10.18632/aging.204515)
Supplement: Supplementary Figures [file aging-15-204515-s001.pdf]

SUPPLEMENTARY FIGURES

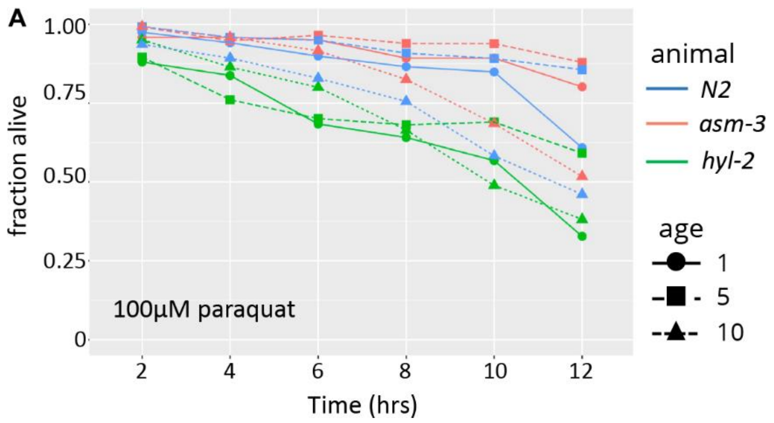

Supplementary Figure 1. *hyl-2*/ceramide synthase animals show poor response to paraquat. (**A**) Survival of 1-, 5-, and 10-day old N2 (blue), *asm-3* (red), and *hyl-2* (green) worms exposed to 100µM paraquat. Worms were treated with paraquat in 96 well plates, and response to agitation was recorded every two hours to determine survival. Each group was done in 6 replicates of 10 worms each.

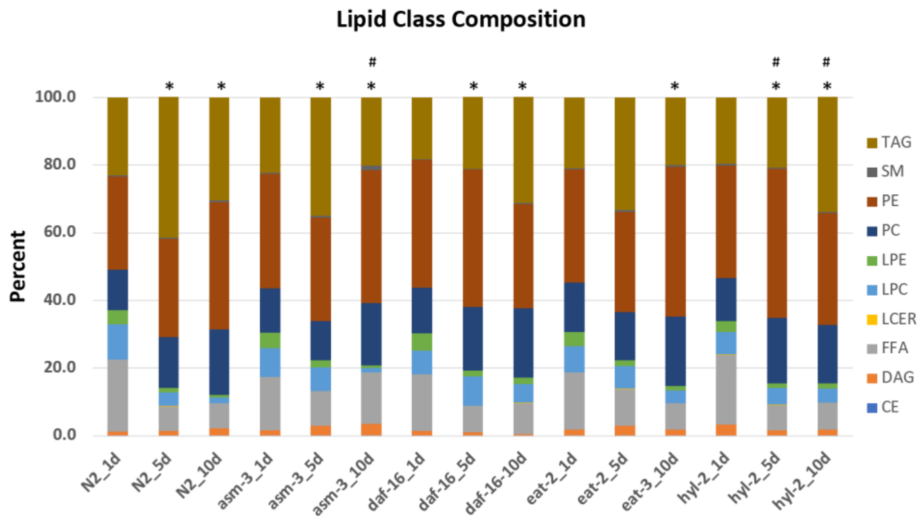

Supplementary Figure 2. Lipid class composition changes as animals age. The percentages were calculated as the proportion of the total lipids (nmol/g of worm tissue (µM)) in each class divided by total lipids in the group. The percentages were then compared to the 1-day N2 animals (* indicates $p<0.05$ compared to N2_1d). Each 5- and 10- day was also compared to the 1-day group for each strain (# indicates $p<0.05$ compared to 1d counterpart). Comparisons were performed using Chi Sq analyses.

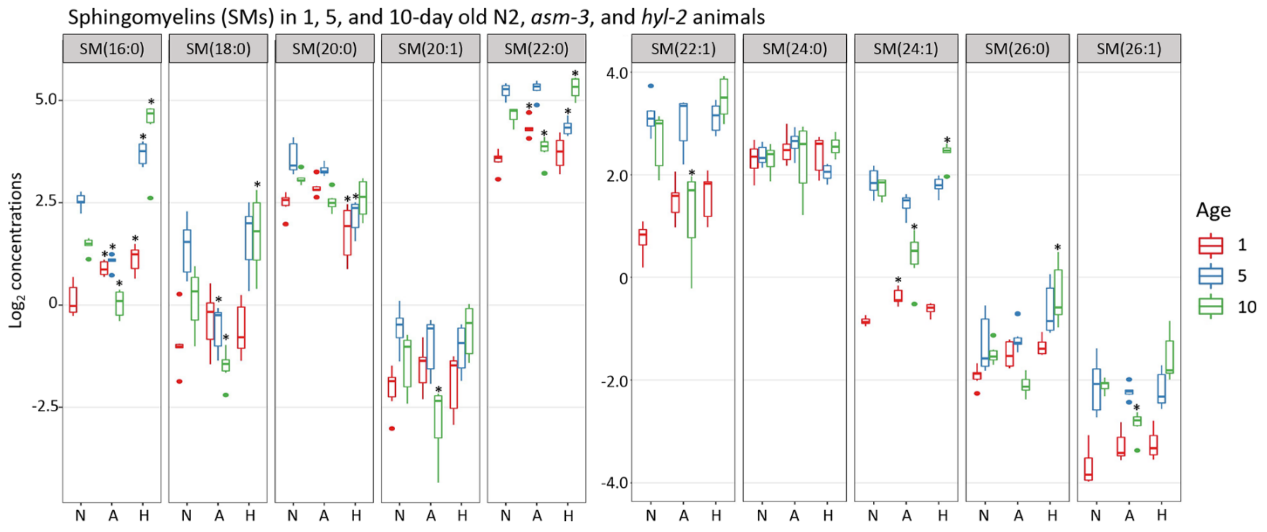

Supplementary Figure 3. Sphingomyelin changes by age in sphingolipid mutants. The boxplots show log2 concentrations of respective sphingolipids in N2 (N), *asm-3*/acid sphingomyelinase (A) and *hyl-2*/ceramide synthase (H) animals. For sphingomyelins, the lipid ID does not include the 18:1 fatty acid chain, and LIPIDMAPS nomenclature is reported in Supplementary table 11. For all, n=6 and * indicates $p<0.05$ compared only to N2 animals of the same age (ANOVA and Tukey-HSD tests).

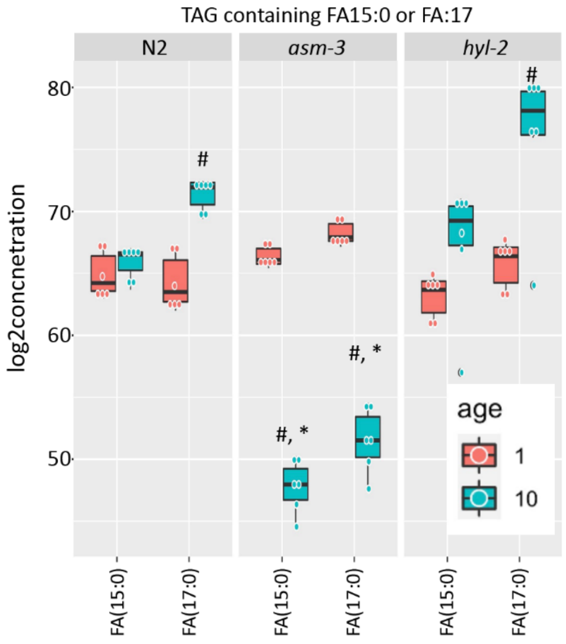

Supplementary Figure 4. Triacylglyercols containing FA15:0 and FA17:0 are increased in asm-3/acid sphingomyelinase mutants. Total Log_2 concentrations of TAG containing FA(15:0) and FA(17:0) chains were summed for 1- and 10-day old N2, *asm-3*, and *hyl-2* animals (n=6 for each group). When comparing N2 and asm-3, there was a significant difference between TAGs containing FA15:0 and FA17:0, and pairwise analyses show significant differences between 10-day concentrations ($p<0.000001$ for FA15:0 and $p<0.000001$ for FA17:0) but not 1-day concentrations. For comparisons between N2 and hyl-2, there was also a significant difference between N2 and hyl-2 animals, but pairwise analysis did not show significant differences between 10-day concentrations ($p=0.93$ for FA15:0 and $p=0.11$ for FA17:0). For all, $^{\#}$indicates $p<0.05$ compared to 1-day counterpart of the same genotype. *indicates $p<0.05$ compared to N2 counterpart of the same age.
