# Supplementary material for: The lipidomes of C. elegans with mutations in asm-3/acid sphingomyelinase and hyl-2/ceramide synthase show distinct lipid profiles during aging
Source: Aging (Albany NY). 2023 Feb 13;15(3):650–74. doi: 10.18632/aging.204515 (PMC9970312; doi:10.18632/aging.204515)
Supplement: Supplementary Table 1 [file aging-15-204515-s002.pdf]

SUPPLEMENTARY TABLE

Supplemental Table 1. Primer sequences for genes used in qPCR.

Gene	Primer sequence (3' - 5')
elo-1	
Forward	CAGTTGGCATTGGTGAAGTGC
Reverse	CCAGGATTCAACAGATACGGA
elo-2	
Forward	GGGTTTGTCGCTTCCTACTG
Reverse	TCAGAGTATGTGACTACTGCG
elo-5	
Forward	ATGCACTGGTACCATCACGC
Reverse	ATGGCAACTGCGAATTGAACC
elo-6	
Forward	AACCATTTGATCTCACGGGACC
Reverse	CACGGCTGAAGAACTCGTGG
fat-1	
Forward	CTTCACCATGCTTTCACCAACC
Reverse	CCGAATAAAGTGTACACTGGG
fat-4	
Forward	GGAGAAGTTTGCATTGAGCTCG
Reverse	TGTGTCGTGGCATCGTTGG
fat-6	
Forward	TCTGATCTCTTGAGTGACCC
Reverse	ATCCGAAATAGTGAGCAGCG
fat-7	
Forward	ACGGCCGTCTTCTCATTTGCT
Reverse	TGGCAACGATGATCACGAGC
cup-16	
Forward	TCGAAGAGAGCACTTAACGGC
Reverse	GCAGATGTTTCAGCGCCTATT
rpl-2	
Forward	TGTGGAGCTAAGGCTCAAATC
Reverse	CGTTGCAGATGGTGGTTCCTT
hyl-2	
Forward	GACCAGAAGGGGAACACTGG
Reverse	CGAGAGCAATGTAGAATCCTCC
sptl-1	
Forward	GAAGAGGAGTCACCGAGCA
Reverse	CCGTTGCAAGAAGAGGTGG
sphk-1	
Forward	ATTCGCAGCTGCAATCTCCG
Reverse	GAATCTGACACGGATCCATTCG
rpl-2	
Forward	TGTGGAGCTAAGGCTCAAATC
Reverse	CGTTGCAGATGGTGGTTCCTT
fat-2	
Forward	GGATATTGAGGTCTACGAAGC
Rreverse	AAGTGATGGGCGACGTGAC
