# Supplementary material for: The lipidomes of C. elegans with mutations in asm-3/acid sphingomyelinase and hyl-2/ceramide synthase show distinct lipid profiles during aging
Source: Aging (Albany NY). 2023 Feb 13;15(3):650–74. doi: 10.18632/aging.204515 (PMC9970312; doi:10.18632/aging.204515)
Supplement: Supplementary Table 6 [file aging-15-204515-s007.pdf]

Supplemental Table 6. Lipid fold changes between 10-day N2 and 10-day eat-2 animals.

Lipid	LogFC	P.Value	FDR
TAG44:1-FA14:1	-5.05071	3.27E-22	1.15E-19
TAG46:3-FA14:0	-3.11684	0.00000000000068	0.0000000000144
TAG45:0-FA15:0	-2.75904	2.37E-24	1.66E-21
TAG46:2-FA18:2	-2.73781	0.000000000000293	0.00000000000684
TAG44:0-FA12:0	-2.72853	0.00000000717	0.0000000529
TAG50:2-FA20:2	-2.67452	1.62E-20	2.84E-18
TAG46:0-FA18:0	-2.66131	0.0000000038	0.0000000296
TAG42:0-FA14:0	-2.65593	0.000276	0.000759
TAG48:4-FA14:1	-2.5399	0.0000000000000000196	0.00000000000000132
TAG50:4-FA14:1	-2.51176	1.06E-18	0.0000000000000000932
TAG46:1-FA14:1	-2.50983	1.31E-19	0.0000000000000000173
TAG46:0-FA12:0	-2.46487	0.00000682	0.000027
TAG52:2-FA20:2	-2.3372	7.3E-19	0.0000000000000000731
TAG44:3-FA18:2	-2.3253	0.000000872	0.00000413
TAG48:3-FA18:3	-2.3128	0.00000000000125	0.0000000000237
TAG47:0-FA15:0	-2.29485	1.48E-19	0.0000000000000000173
TAG48:2-FA18:2	-2.2154	0.0000000000000000539	0.0000000000000027
TAG46:2-FA14:1	-2.2092	0.0000000000000000208	0.00000000000000132
TAG44:1-FA18:1	-2.19892	0.00000000000134	0.0000000000247
TAG50:1-FA20:1	-2.19501	0.0000000000000000525	0.0000000000000027
TAG46:3-FA18:2	-2.18545	0.0000000000000115	0.000000000000424
TAG50:5-FA18:2	-2.16965	0.0000000114	0.0000000813
TAG47:0-FA17:0	-2.16637	6.85E-18	0.000000000000000534
TAG45:1-FA15:0	-2.15197	0.000000000000000234	0.000000000000011
TAG42:1-FA14:0	-2.10766	0.0000854	0.000259
TAG48:1-FA14:1	-2.09764	0.0000000000000000525	0.0000000000000027
TAG46:1-FA16:1	-2.08682	0.0000000000000133	0.000000000000465
TAG47:2-FA18:2	-2.05378	0.00000000000000278	0.000000000000122
LPE(20:4)	2.002917	0.00000000413	0.0000000318
CE(20:4)	2.058696	0.00000147	0.00000647
PE(18:1/18:4)	2.116137	0.00000333	0.000014
LPC(20:0)	2.15351	0.003403	0.007454
PE(18:0/18:4)	2.728121	0.00000000124	0.0000000108
