# Supplementary material for: The lipidomes of C. elegans with mutations in asm-3/acid sphingomyelinase and hyl-2/ceramide synthase show distinct lipid profiles during aging
Source: Aging (Albany NY). 2023 Feb 13;15(3):650–74. doi: 10.18632/aging.204515 (PMC9970312; doi:10.18632/aging.204515)
Supplement: Supplementary Tables 10 and 11 [file aging-15-204515-s011.pdf]

Supplemental Table 10. Relative gene expression and fold change values from RT-qPCR analyses of 10-day and 1-day WT, asm-3 and hyl-2 animals.

		1day			10day			10day/1day		
Gene	Animal	2ddCT	SEofdiff	p value (vs N2)	2ddCT	SE	p value (vs N2)	FC	log2FC	p value
rpl-2	N2	1	0.37	n/a	1.12	0.35	n/a	1.32	0.17	0.998
rpl-2	hyl-2	1.21	0.28	0.984	1.28	0.38	0.997	1.03	0.08	1
rpl-2	asm-3	0.94	0.4	1	1.56	0.69	0.865	1.74	0.73	0.542
elo-5	N2	1	0.75	n/a	0.24	0.5	n/a	-4.13	-2.05	0.005
elo-5	hyl-2	2.05	0.54	0.902	0.42	0.33	0.149	-4.92	-2.3	0.002
elo-5	asm-3	1.22	0.61	0.232	0.54	0.75	0.496	-2.25	-1.17	0.144
elo-6	N2	1	0.7	n/a	0.39	0.51	n/a	-2.57	-1.36	0.081
elo-6	hyl-2	1.74	0.48	0.501	0.34	0.51	0.997	-5.13	-2.36	0.002
elo-6	asm-3	1.41	0.41	0.867	0.7	0.64	0.44	-2.01	-1.01	0.276
elo-1	N2	1	0.33	n/a	0.96	0.65	n/a	-1.04	-0.06	1
elo-1	hyl-2	0.71	0.27	0.696	1.23	0.63	0.881	1.73	0.79	0.245
elo-1	asm-3	1.14	0.48	0.993	1.47	0.72	0.488	1.29	0.37	0.873
elo-2	N2	1	0.35	n/a	0.44	0.63	n/a	-2.27	-1.18	0.161
elo-2	hyl-2	0.45	0.39	0.182	1.01	1	0.15	2.24	1.16	0.17
elo-2	asm-3	2	0.49	0.293	1.03	0.72	0.142	-1.95	-0.96	0.327
fat-1	N2	1	0.37	n/a	1.12	0.35	n/a	1.12	0.17	0.923
fat-1	hyl-2	1.21	0.28	0.753	1.28	0.38	0.995	1.06	0.08	0.43
fat-1	asm-3	0.94	0.4	0.998	1.56	0.69	0.735	1.66	0.73	1
fat-2	N2	1	0.67	n/a	0.63	0.71	n/a	0.63	-0.67	1
fat-2	hyl-2	1.18	0.67	0.683	0.86	0.71	0.865	0.73	-0.46	0.998
fat-2	asm-3	0.69	0.58	0.998	1.25	0.64	0.361	1.81	0.86	0.667
fat-4	N2	1	0.41	n/a	0.81	0.48	n/a	-1.23	-0.3	0.936
fat-4	hyl-2	1.29	0.49	0.863	0.91	0.5	0.996	-1.43	-0.51	0.637
fat-4	asm-3	0.8	0.45	0.803	1.72	0.66	0.056	2.16	1.11	0.049
fat-6	N2	1	0.7	n/a	0.44	0.66	n/a	0.44	-1.18	0.669
fat-6	hyl-2	1.37	0.75	0.83	0.87	0.91	0.316	0.64	-0.66	0.993
fat-6	asm-3	0.8	0.77	1	1	0.93	0.616	1.25	0.32	1
fat-7	N2	1	0.69	n/a	0.04	0.63	n/a	0.04	-4.64	0
fat-7	hyl-2	0.81	0.69	0.968	0.01	0.79	0	0.01	-6.34	0
fat-7	asm-3	0.46	0.72	0.516	0.03	0.8	0.201	0.07	-3.94	0
asm-3	N2	1	0.69	n/a	0.2	1.1	n/a	-4.91	-2.3	0.226
asm-3	hyl-2	2.33	0.72	0.762	0.07	1.2	0.666	-33.51	-5.07	0.01
asm-3	asm-3			n/a			n/a			
hyl-2	N2	1	0.77	n/a	3	0.93	n/a	3	1.58	0.241
hyl-2	hyl-2			n/a			n/a			
hyl-2	asm-3	1.29	0.66	0.992	4.13	0.84	0.979	3.2	1.68	0.196
cup-16	N2	1	0.4	n/a	11.26	0.87	n/a	11.26	3.49	0
cup-16	hyl-2	0.76	0.59	0.949	17.35	0.77	0.764	22.94	4.52	0
cup-16	asm-3	0.81	0.53	0.984	22.01	0.79	0.366	27.22	4.77	0
sptl-1	N2	1	0.94	n/a	1.17	1.13	n/a	1.17	0.23	1
sptl-1	hyl-2	1.42	0.93	0.987	0.99	1.04	1	0.7	-0.52	0.985
sptl-1	asm-3	1.46	0.62	0.982	2.51	0.9	0.747	1.73	0.79	0.919
sphk-1	N2	1	0.59	n/a	1.08	0.8	n/a	1.077	0.11	1
sphk-1	hyl-2	1.09	0.78	1	1.57	0.9	0.919	1.441	0.53	0.928
sphk-1	asm-3	1.11	0.64	1	3.25	0.79	0.116	2.915	1.54	0.133

Supplementary Table 11. LIPIDMAPS and common names of sphingolipids examined.

	ID	HMDB	LIPIDMAPS	Common name
1	SM(14:0)	HMDB12097	LMSP03010028	SM(d18:1/14:0)
2	SM(16:0)	HMDB10169	LMSP03010003	SM(d18:1/16:0)
3	SM(18:0)	HMDB01348	LMSP03010001	SM(d18:1/18:0)
4	SM(18:1)	HMDB12101	LMSP03010029	SM(d18:1/18:1(9Z))
5	SM(20:0)	HMDB12102	LMSP03010005	SM(d18:1/20:0)
6	SM(20:1)	-	LMSP03010059	SM(d18:1/20:1)
7	SM(22:0)	HMDB12103	LMSP03010006	SM(d18:1/22:0)
8	SM(22:1)	HMDB12104	LMSP03010072	SM(d18:1/22:1)
9	SM(24:0)	HMDB11697	LMSP03010008	SM(d18:1/24:0)
10	SM(24:1)	HMDB12107	LMSP03010007	SM(d18:1/24:1(15Z))
11	SM(26:0)	HMDB11698	LMSP03010010	SM(d18:1/26:0)
12	SM(26:1)	-	LMSP03010009	SM(d18:1/26:1(17Z))
